# The Complexity of Human Walking: A Knee Osteoarthritis Study

**DOI:** 10.1371/journal.pone.0107325

**Published:** 2014-09-18

**Authors:** Margarita Kotti, Lynsey D. Duffell, Aldo A. Faisal, Alison H. McGregor

**Affiliations:** 1 Biodynamics Laboratory, Division of Surgery, Department of Surgery and Cancer, Faculty of Medicine, Imperial College London, London, United Kingdom; 2 Brain Behaviour Laboratory, Department of Bioengineering, Imperial College London, London, United Kingdom; 3 Department of Computing, Imperial College London, London, United Kingdom; 4 MRC Clinical Sciences Centre, Faculty of Medicine, Imperial College London, London, United Kingdom; University of Texas Health Science Center at San Antonio, Research Imaging Institute, United States of America

## Abstract

This study proposes a framework for deconstructing complex walking patterns to create a simple principal component space before checking whether the projection to this space is suitable for identifying changes from the normality. We focus on knee osteoarthritis, the most common knee joint disease and the second leading cause of disability. Knee osteoarthritis affects over 250 million people worldwide. The motivation for projecting the highly dimensional movements to a lower dimensional and simpler space is our belief that motor behaviour can be understood by identifying a simplicity via projection to a low principal component space, which may reflect upon the underlying mechanism. To study this, we recruited 180 subjects, 47 of which reported that they had knee osteoarthritis. They were asked to walk several times along a walkway equipped with two force plates that capture their ground reaction forces along 3 axes, namely vertical, anterior-posterior, and medio-lateral, at 1000 Hz. Data when the subject does not clearly strike the force plate were excluded, leaving 1–3 gait cycles per subject. To examine the complexity of human walking, we applied dimensionality reduction via Probabilistic Principal Component Analysis. The first principal component explains 34% of the variance in the data, whereas over 80% of the variance is explained by 8 principal components or more. This proves the complexity of the underlying structure of the ground reaction forces. To examine if our musculoskeletal system generates movements that are distinguishable between normal and pathological subjects in a low dimensional principal component space, we applied a Bayes classifier. For the tested cross-validated, subject-independent experimental protocol, the classification accuracy equals 82.62%. Also, a novel complexity measure is proposed, which can be used as an objective index to facilitate clinical decision making. This measure proves that knee osteoarthritis subjects exhibit more variability in the two-dimensional principal component space.

## Introduction

The aim of this study is to check whether the redundant dimensionality of the human biomechanical system can be effectively reduced by projection into a low principal component (PC) space. In the later space it is proven that the patterns produced by normal subjects and pathological subjects that suffer from knee osteoarthritis (OA), are still identifiable. A challenge in analysing gait patterns is that, as a form of behaviour, it exhibits high variability [1]. Both sensory inputs and motor outputs are subjected to noise and uncertainty [2] [3].

Additionally, movement analysis is extremely complex since the musculoskeletal system has over 600 degrees of freedom. We assume that the design of our muscoloskeletal system is redundant and, as a result of, this the central nervous system has several options when generating movement for a specific task. In [4] it is indicated that muscular redundancy is necessary, however the idea of redundancy still greatly increases the complexity incurred when generating movement. Movement data is inherently variable both within subjects (across trials) as well as across subjects [5]. Most traditional motion analysis methods simply average away the variability in the data to obtain a clear readout of an underlying mechanism. This dismisses a lot of the obtained data implying that features buried in the structure of variability of behavioural data are lost. In contrast, we embrace here the variability of the data, as we hypothesis the structure of variability provides insight into the underlying mechanisms. The novelty of our idea is that instead of averaging variability out we take the view that the structure of variability may contain valuable information about the task being performed [5]. We examine whether motor behaviour can be understood by identifying a simplicity, through projection to a low PC space, which may reflect upon the underlying mechanism [6], [7]. Previous research has verified the existence of stereotypical patterns of correlation between joints of the fingers during everyday tasks [6] [8] [9]; or even recognising motion segments from the whole body [10]. Here, we confine ourselves to human walking, which is highly complex and exhibits long-range correlations and self-similarity, although there are differences between normal and pathological gait [11], [12].

The reason why we chose OA is that it is a widespread joint disease affecting many individuals, it is known to alter gait and function and as such is an ideal condition to test the proposed machine learning protocol for detecting patterns that are characteristic of changes from normality. It is also worth mentioning that although altered gait profiles have been linked with OA, it is unknown if abnormal gait is a cause or effect of the disease [13]. OA is the most widespread joint disease; and this is forecast to increase with the rapidly ageing population. OA leads to pain, stiffness, weakness, joint instability, and reduced range of motion. It ranks as the 2nd cause of disability [14], leading to 171 million years of life lived with disability [15]. Not surprisingly OA is now recognized as the fastest growing major health condition. Current estimates project that 40% of people over 70 years of age will suffer with knee OA, experiencing severe pain, and limited joint motion; with 25% of this group experiencing a major impact on daily activities [16]. Of greater concern is the fact that patient numbers are predicted to more than double in the near future, as the ageing population expands. In the UK, for example, a twofold increase is predicted by 2030 [17]. Given its prevalence, it is not surprising that OA poses a huge socioeconomic burden, both in the UK and worldwide. More than 1 million adults consult their GP each year with OA in UK alone [18]. Currently, diagnosis is based on radiographic findings [19], which implies an advanced stage of knee OA. The gold standard is MRIs for identification of changes in cartilage but these are expensive and usually clinicians resort to them until symptoms are severe and restricting. Although imaging is frequently used it is commonly acknowledged that imaging and patient reporting of pain and loss of function do not always align with some subject reporting high pain and reduced functionality, with limited evidence of joint degeneration on imaging. Early problem identification could prove to be beneficial, since late interventions, such as total knee arthroplasty although successful in removing pain, may lead to compromised functions, leaving recipients dissatisfied [20] [21]. Although joint replacements are considered successful they do have a finite lifespan and frequently require replacing within 10 years of initial surgery. With people living longer this poses a problem and the ideal would be to develop interventions to delay joint deterioration and the need for replacement such that implants would last the lifetime of the recipient [22].

The short-term purpose of this study is to propose a holistic framework that can automatically detect patterns in the walking data by projecting them into a low-dimensional PC space. The long-term purpose of this study is to offer clinicians an automated tool that can support them with their clinical decisions by calculating the probability that a patients suffers from knee OA. To achieve this, we have collected gait patterns from 180 subjects, 47 of which reported that they suffer from knee OA. The parameters examined here are the ground reaction forces (GRFs) recorded in the vertical, anterior-posterior, and medio-lateral axis. To analyse the aforementioned data we employed machine learning techniques, since the latter may reveal implicit information that is hidden in the data but cannot be revealed by human eye. Machine learning offers novel tools that can expand and augment classical statistics and hypothesis testing. Machine learning tries to find patterns in the data and discover the hidden relationship among several parameters. Therefore we apply Probabilistic Principal Component Analysis (PPCA) to recover the variability structure of the data and show that the variability signature extracted carries predictive power for OA detection from force plate data. Moreover it allows us an objective definition of how complex the force plate time series are that are generated by walkers. Thus, we can compare how complex the force patterns between healthy and OA patients are and we find this method to be able to efficiently detect the knee OA subjects in our population.

In more detail, PPCA captures the main components of motion, that account for most of its variability. Those components are then used in order to build motion models. Specifically, two models are built to explore our understanding of the gait patterns: one of them for the gait patterns produced by control subjects and a second one for the gait patterns of knee OA sufferers. The models are multidimensional Gaussians and in order to assess whether a pattern is derived by a normal or a knee OA subject, a Bayes classifier is utilised. A Bayes classifier provides two probabilities: the probability that the subject comes from the control population and the probability that the subject suffers from knee OA. In short, this approach aims to automatically detect knee OA, while revealing the fundamental structure of motion.

Previous biomedical studies on discriminating subjects with knee OA vs. normal subjects using machine learning are available in the literature. For example the sagittal/frontal/transverse plane range of motion along with the maximum of the vertical GRF and cadence are used to discriminate between 15 normal and 15 knee OA subjects, using the Dempster-Shafer theory of evidence in [23]. In another study [24], knee flexion angle, flexion moment, and adduction moment for 50 patients with end-state knee OA and 63 aged-matched asymptomatic control subjects are analysed via principal component analysis and discriminant analysis cycle. Important differences with respect to knee OA included smaller knee flexion moments during stance, larger knee adduction moments during the stance phase of the gait cycle, and smaller knee exion angle ranges of motion throughout the gait cycle.

In our work, we reduced the dimensionality of the initial GRF patterns via PPCA, a procedure which allowed us to identify the underlying structure of the walking patterns that supports the view that the cortex organises behaviour in a low-dimensional manner, although the muscoloskeletal system is redundant. Next, by exploiting the covariance as it was calculated by PPCA, we created two multivariate Gaussian models of human locomotion. If we project from the initial 606 dimensional space to a 36 dimentional space, an accuracy of 82.62% in differentiating between subjects that are normal and those that suffer from knee OA is achieved.

## Materials and Methods

### Subjects

A total of 180 subjects participated in this study, 47 of which were diagnosed with OA. All control subjects were recruited from staff and students at Charing Cross Hospital and posters circulated in hospitals/gyms/local health centres. OA subjects were recruited from clinics in Charing Cross Hospital and local district hospitals. For those subjects OA was diagnosed by their clinicians (GPs or orthopaedics). For imaging verification, a multitude of techniques were used, such as MRIs, x-rays, or CTs. Concerning the side of the pathology, OA could affect either medial or lateral tibiofemoral compartment or it could be patellofemoral or a combination of these. Subjects were excluded from the study if they reported rheumatoid or other systemic inflammatory arthritis, morbid obesity (Body Mass Index >35 kg/m2) or had undergone previous surgical treatment for knee OA, besides arthroscopy. More demographic details of the recruited subjects, such as age, height, weight, etc, are demonstrated in Table 1.

**Table 1:** Mean value and standard deviation about the age, height, weight, BMI, sex, and pain (as assessed by the KOOS score) and the number of subjects that have experienced a surgery or an injury for both the control and the knee OA subjects.

	no knee OA	knee OA
	(133 subjects)	(47 subjects)
Age (years)	45.0 (16.5)	58.1 (12.7)
Height (mm)	1714.6 (102.2)	1695.8 (113.2)
Weight (kg)	69.2 (12.4)	76.2 (14.4)
BMI (kg/mm^2^)	23.4 (2.9)	26.381(3.325)
Male/Female	66/67	22/25
Previous Injury	35.3%	46.8%
Previous Surgery	23.3%	66.0%
Pain (KOOS)	90.9 (13.4)%	60.8 (18.9)%

Demographic details of the subjects

### Ethics statement

Ethical approval for this study was obtained from the South West London Research Ethics Committee and all subjects provided written informed consent. The individual that appears in this manuscript has given written informed consent (as outlined in PLOSconsent form) to publish these case details.

### Data acquisition

**Figure 1:**
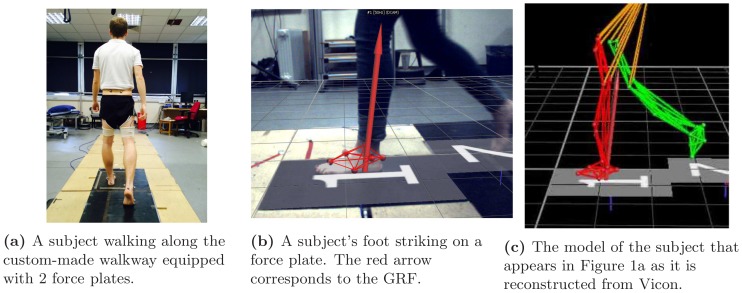
Data capturing. Figure 1a is the real, lab-based environment, Figure 1c is the computer reconstruction, whereas Figure 1b an overlay of the two.

Subjects were asked to walk at their normal speed along a 6 m walkway embedded with two force plates (Kistler Type 9286B, Kistler Instrumente AG, Winterthur, Switzerland). A picture of the walkway along with a subject walking can be seen in Figure 1a. The individual in this manuscript has given written informed consent (as outlined in PLOSconsent form) to publish these case details. Each subject was barefoot and was asked to walk along the walkway a minimum of five times. Trials with no clean force plate strike were excluded. A maximum of three trials where the subject cleanly struck the force plate were recorded for the left and right foot. Since the 180 subjects provided 1–3 trials, a total of 532 trials were available. The signals from the force plates were recorded using an analogue signal data acquisition card provided with the Vicon system (Vicon Motion Systems Ltd, Oxford, UK) and the Vicon Nexus software at a sampling rate of 1000 Hz. The GRF corresponds to the red arrow depicted in Figure 1b over the real world image and on Figure 1c over the Vicon reconstruction.

Data comprised of GRFs for all three planes: vertical, medio-lateral, and anterior-posterior. GRF data was normalised to the subjects' body weight (N/kg), and was time-normalised to the entire gait cycle using linear interpolation. This way, we obtained 101 samples per gait cycle. Given that all three axes are considered for the GRF and that we consider both knees for each subject, the gait pattern for each trial has a total length of 3×2×101 = 606 samples.

## Results

### Data pre-processing

**Figure 2:**
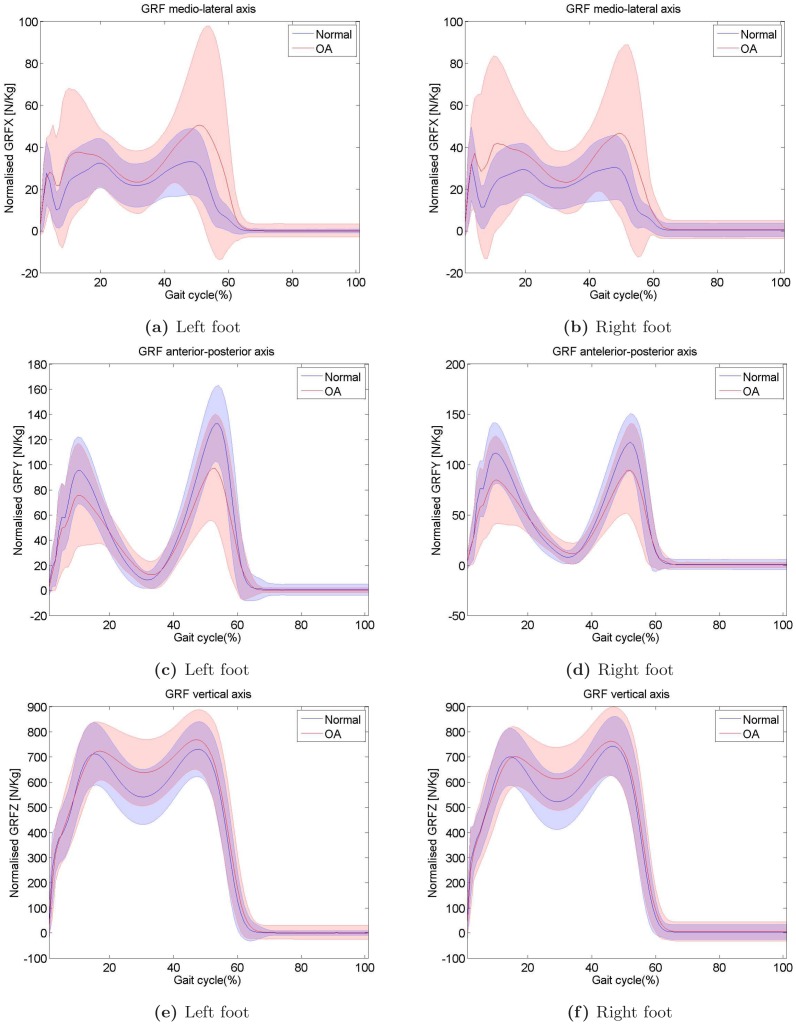
The blue curve corresponds to the mean GRF curve, whereas the blue shaded region indicates the precision of plus minus one standard deviation. Accordingly, the foot which has knee OA is depicted in red.

Before analysing the data, we visualised them for each axis and for each leg separately. In Figure 2 the medio-lateral (GRF-X), anterior-posterior (GRF-Y), and vertical (GRF-Z) axes are depicted both for normal as well as for knee OA subjects. The blue curve corresponds to the mean GRF curve for the normal knee, whereas the blue shaded region indicates the aforementioned mean plus minus one standard deviation. Accordingly, the red curve corresponds to knee OA. It can be seen that the GRFs for the knee OA subjects exhibit higher variability that than of normal subjects, for which GRFs are more consistent. In the anterior posterior axis, knee OA subjects exhibit lower GRFs, whereas for the two other axes, it is the normal subjects that exert lower forces. Finally, it is also evident from Figure 2 that GRFs developed over the two legs are not strictly symmetrical. This adds to the complexity of the problem and contributes to the belief that there are random signal disturbances of our nervous-system function which are responsible for coordinating motion [5].

**Figure 3:**
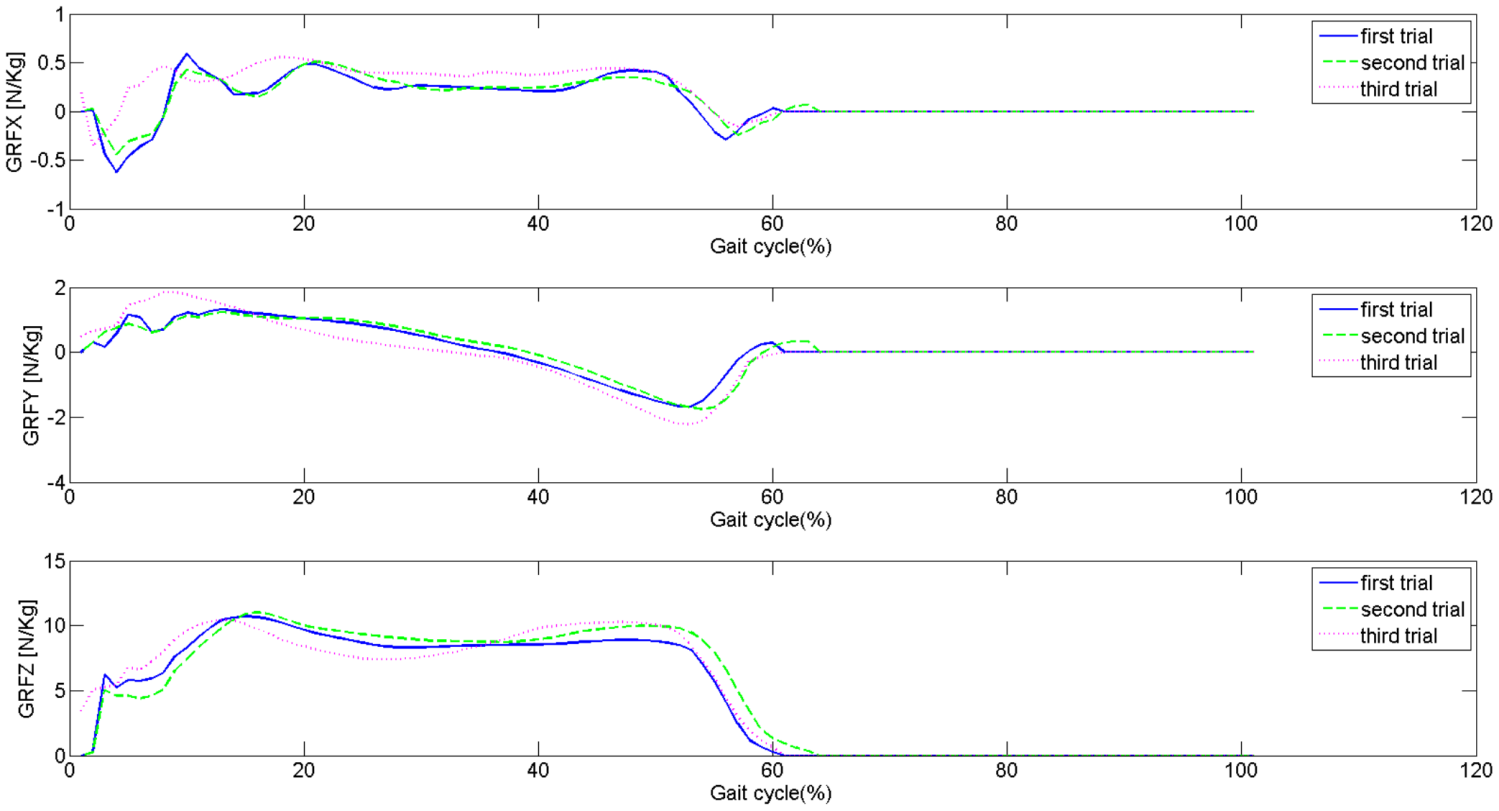
GRFs for an random indicative subject.

As already discussed, human behavioral data exhibit high variability [25] [26]. Regrading inter-subject variability, it is easy to see in Figure 2 that subjects walk in a different manner, i.e. that the inter-subject variability is high, as indicated by the width of ±1 standard deviation, that is the shaded area. The intra-subject variability is depicted in Figure 3, where 3 trials for one indicative subject are depicted.

**Figure 4:**
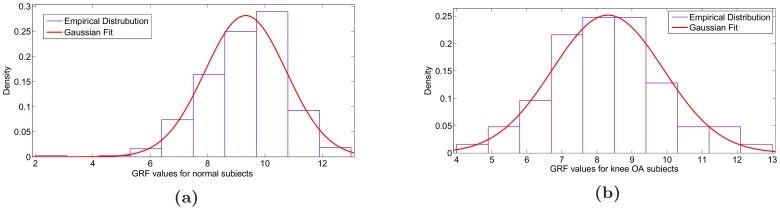
The goodness of fit of a Gaussian distribution to the actual empirical distribution of the GRFs patterns for (a) normal subjects and (b) knee OA subjects. Probability distributions over GRF patterns. Solid red lines is Gaussian distributions with mean and standard deviation matched to the empirical GRFs histograms. The data and the matching Gaussian distributions appear as bell-shaped.

Finally, it is useful to verify that the collected actual empirical GRFs are indeed Gaussian distributed. This is because PPCA exploits a Gaussian latent variable model and can be utilised as a general Gaussian density model [27]. Also, the Bayesian classifier used in this work, assumes that the samples of each class follow the Gaussian distribution. The collected GRF histograms are depicted in Figures 4a-4b along with the fitted Gaussian distributions. As verified by the aforementioned Figures, the empirical distributions are well-fitted with a Gaussian distribution. The empirical pdf histogram is depicted in blue. The theoretical distribution pdf fitted on the empirical data histogram is depicted by a solid red line. Although the GRF statistics for walking are indeed Gaussian distributed, our method would work irrespective of the actual empirical data distribution, as we simply use the amount of variance explained by each PC (and compute these for all dimensions) as characteristic to measure and distinguish walking patterns.

#### Probabilistic principal component analysis

PPCA reduces the set of correlated data to a set of non correlated variables, called principle components. The first component contains the maximum value of the variance (maximum variability of data), followed by the second and so forth to the last PC [28] [27]. Because of that, PPCA has been used to determine the complexity of human walking by reducing the dimensionality of the space (here, a 606 dimensional space) and measuring the amount of variance of the data contained by each of the PCs.

To provide a short overview [27], we denote the observed data, i.e. the GRF patterns by 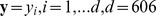, and the significantly lower 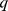 dimensional latent vectors as 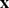. It is true that 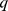 equals to the number of PCs we retain. The following relationship is considered:



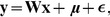
(1)where 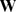 is the matrix that projects 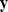 to 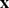; 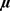 is the mean vector of the model; and 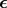 is the model noise. If the noise 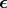 is isotropic Gaussian noise, i.e.,



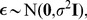
(2)then it holds that



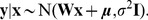
(3)


If we consider that the latent variables also follow a Gaussian distribution, i.e. 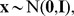 then:



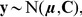
(4)where



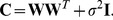
(5)


The Maximum Likelihood Estimators (MLEs) of 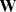 and 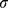 are:



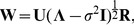
(6)and



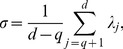
(7)where 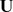 are the principal eigenvectors of the sample covariance matrix of 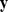, with eigenvalues 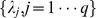 that form the diagonal matrix 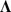, and 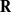 is a rotation matrix.

Here, we compute the covariance matrices 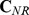 and 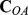 for the normal and for the knee OA model, respectively [29] by utilising PPCA. Those matrices are subsequently used by the Bayes classifier to compute the probabilities that a testing GRF vector 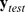 belongs to each distinct class.

#### A new measure of complexity

As a way to quantify the complexity of the ground reaction forces for a given number of PCs, we propose a new measure that we call motion complexity 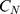.



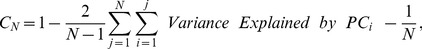
(8)where 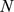 is the total number of PCs we consider. This implies that the lower the value, the higher the complexity.

**Figure 5:**
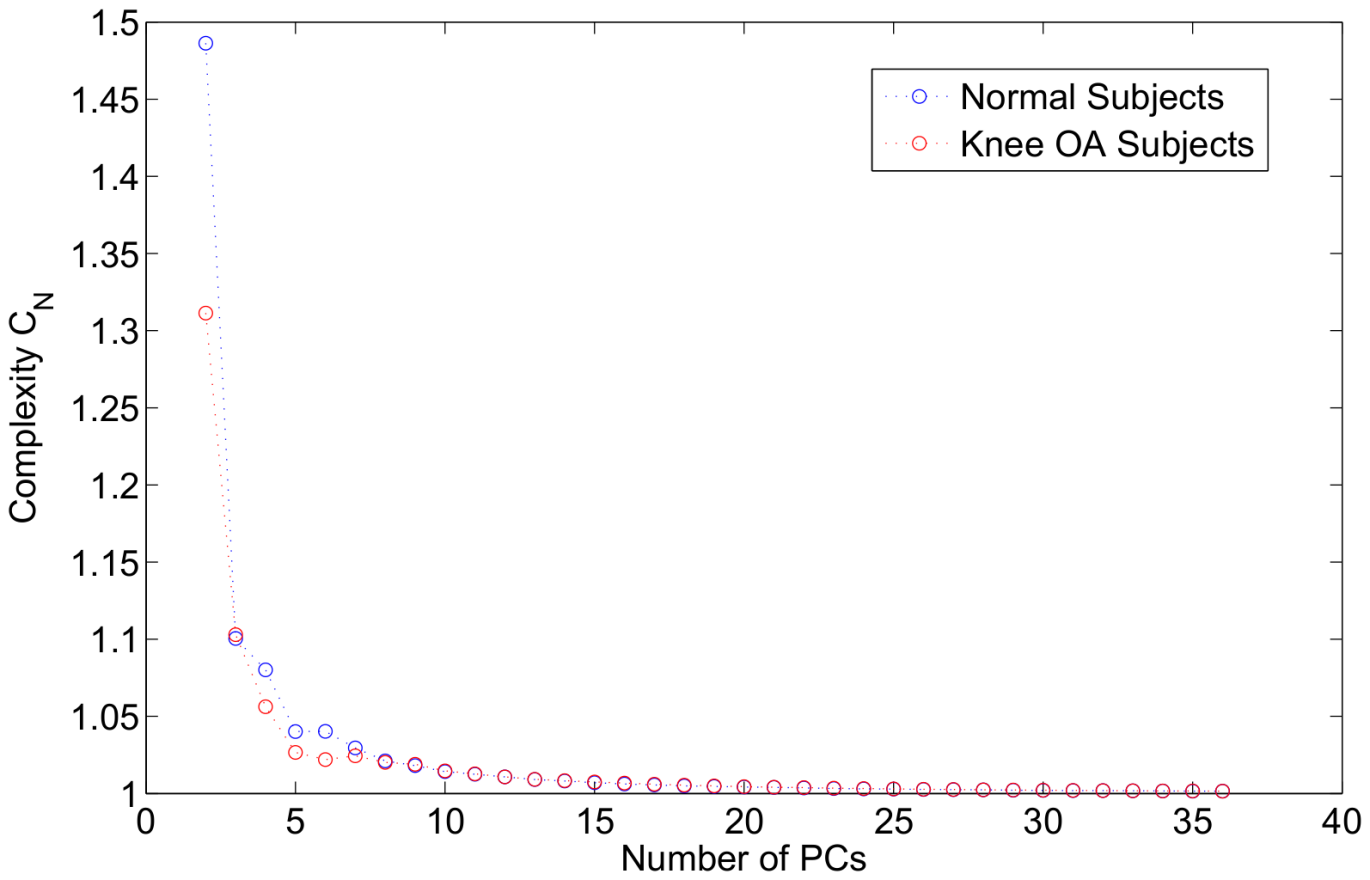
The proposed complexity measure 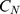 for the first 36 PCs. In the lower PC dimensional space knee OA subjects have a tendency to present lower values.

**Figure 6:**
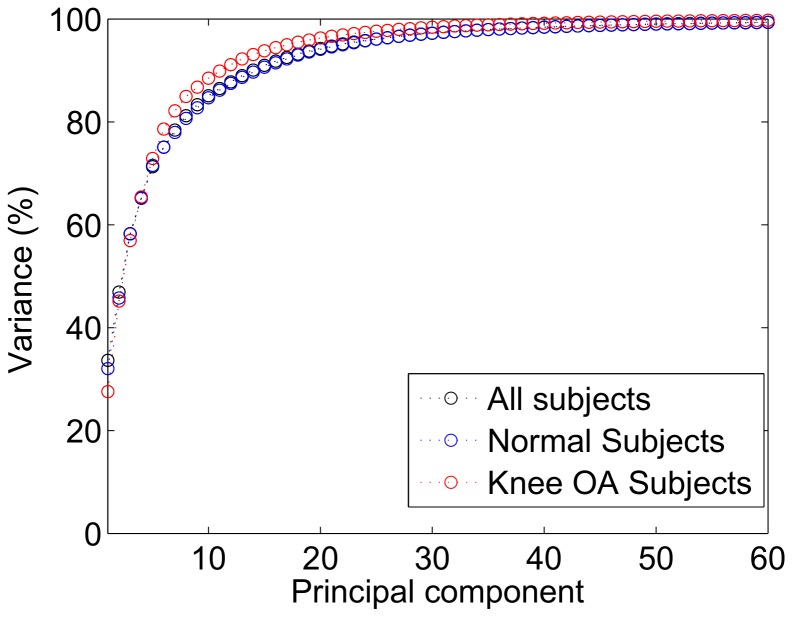
How much variability is explained as a function of the number of the components. The x-axis corresponds to the number of PCs, whereas the y-axis the percentage of the variance of the GRF patterns explained by the respective number of PCs. It is evident that human walking is a complex process, since the slope starts at a low point (1 PC explains just above 30% of the variability of the combined data) and the slope progresses slowly.

With respect to the newly defined complexity measure 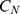, a diagram of how complexity is progressing with respect to the number of PCs can be seen in Figure 5. It can be concluded from the Figure that in the lower PC dimensional space knee OA subjects walk in a more complex manner, whereas when a higher number of PCs is exploited, the complexity between the two groups converges. This is in line with the interpretation of Figure 6, where it is seen that for first 3 PCs the variability explained for knee OA subjects is lower compared to the normal ones. For example, 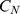 = 1.311 for the knee OA subjects, whereas 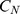 = 1.486 for the normal subjects for the simple case of considering the first two PCs. This way complexity can be thought as a new assessment measure that is calculated after an objective mathematical analysis and can potentially support clinicians when taking decisions.

#### Bayes classifier

Classification of the data is accomplished by means of a Bayes classifier. We consider 2 classes, i.e. healthy subjects vs subjects that suffer from knee osteoarthritis. In the training phase, we estimate the multivariate Gaussian distribution parameters by utilising the 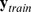 GRF vectors. For each class we estimate the mean vectors 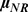 and 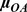, for the normal and the osteoarthretic classes respectively, as well as covariance matrixces 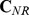 and 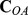 using Eq.(5).

The probability of an observed GRF vector of the test set 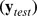 to be derived from a normal walking subject is:



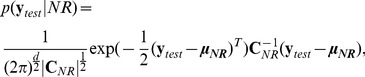
(9)given that 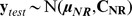. Accordingly, we compute the probability 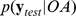 for a subject that suffers from knee OA. The Bayes rule [30] for 2 classes 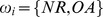, states that 
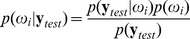
. Accordingly, the Bayes classifier denotes that [31]:



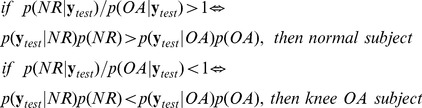
(10)


Figure 6 shows the amount of variance explained in the data versus the number of pricipal components. In this Figure we confine ourselves to the first 60 PCs, since, for the whole dataset, i.e. normal subjects and subjects that have knee OA, over 99% of the variance is explained by those components. The readability of the Figure is significantly decreased if we utilize all 606 PCs. From Figure 6 it can be concluded that the first PC explained just over 33% of the variability of the data, the first 2 PCs explained about 45% of the variance in the data, whereas the explained variance percentage raises to almost 60% for 3 PCs. For the same Figure, it is also evident that if we confine ourselves to a 1-dimensional PC space, then the variance explained of the knee OA subjects is lower that for the normal subjects. However, for as the number of PCs increases, the knee OA subjects exhibit less variance.

**Figure 7:**
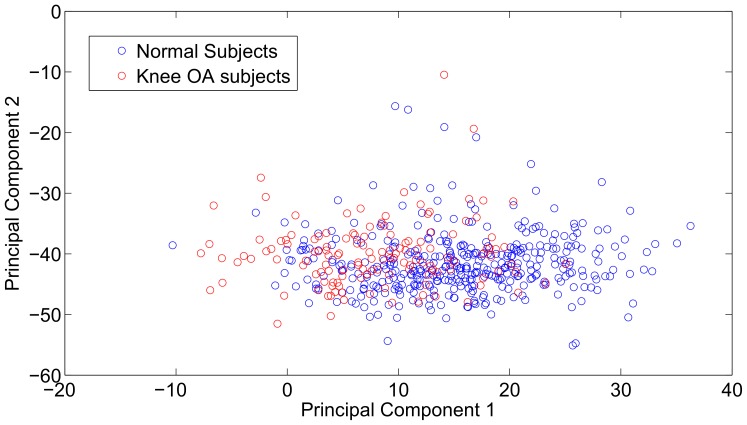
Projection of the GRF patterns in 2-D PC space (i.e. the first two PCs). The two classes are not separable.

The complexity of the structure of human walking can be visualised if we plot the projection of the original GRF patterns in the PC space. In Figure 7 the projection to a 2-dimensional space is demonstrated. To conclude, PPCA on a limited number of steps (1–3 steps) revealed that complex walking patterns restricted on a low dimensional subspace are not easily separable.

**Figure 8:**
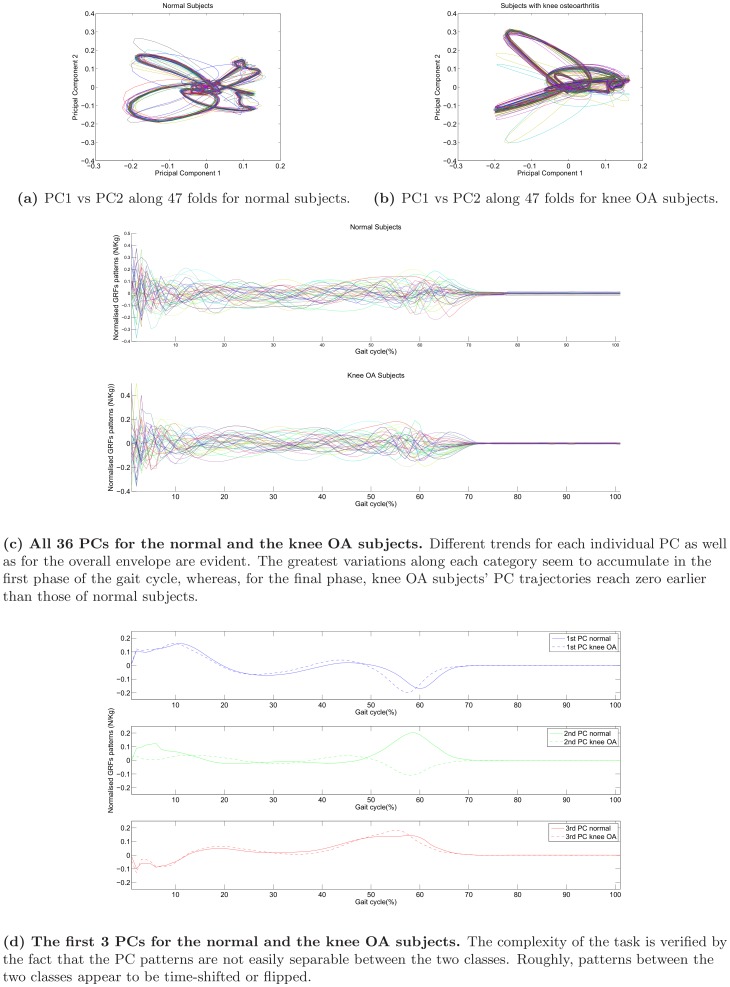
PC visualisations as discriminants of the two classes: normal subjects vs. knee OA subjects.

Aiming to visualise how different are the walking patterns between normal and knee OA subjects, the trajectories of PC 1 vs PC 2 are depicted in Figures 8a-8b for all 47 folds. As explained in detail later on in this Section, a 47-cross validation protocol guarantees that we maximise the number of subject-independent training patterns. Each trajectory corresponds to one fold, since one normal and one osteoarthretic model is built for each fold. It becomes clear from Figures 8a-8b that there is an underlying structure in the GRF patterns and that those patterns are considerably different between the normal subjects and the subjects that have knee osteoarthritis. The greater variability among the normal subjects can be attributed to the fact that it is expected that some subjects may have early signs of knee OA, but were asymptomatic at the time of the study. Thus they cover a higher range of the disease presence and motion patents, compared to the knee OA subjects subset. The latter is more uniform, since all subjects have already been diagnosed with knee OA. Ultimately, the walking trajectories in the PC space exhibit a different structure.

An alternative way to visualise the difference in the structure of the PC space is to depict all PCs for the normal and the knee OA subjects. We decided to utilise 36 dimensions because those explained about 99% of the variance in the data. In Figure 8c, PCs are extracted for one indicative fold of the 47-folds protocol and each PC is depicted with a different color. Since the component subspace is a 36-D basis, PCs are unit vectors. It is clear that PCs are following different trajectories for the two classes. To increase the clarity of the Figure 8c, we focus on the first 3 PCs for which the trajectories for the two classes are demonstrated in Figure 8d. Solid lines stand for the normal subjects, whereas dashed lines indicate the knee OA subjects. Visual inspection verifies that all 3 PCs follow different trajectories over the two classes, whereas the 2nd PC is the most discriminative among the two classes.

Next, we applied a Bayes classifier in order to distinguish the two classes. The experimental protocol is subject-independent. If a subject's trial is included in the training set, then all the trials of this subject are part of the training set and none is used in the test set. Subject-independent systems present several advantages. They are able to handle efficiently an unknown subject [32]. Thus, they are more robust and stable, and demonstrate a better generalization ability than the subject-dependent ones, since they avoid classifier over-fitting. In subject-dependent experimentation it is possible that the classifier may learn special characteristics of the specific subject along with the pathological locomotion patterns. Finally, subject-independent systems are suitable for real-life applications, such as a general practitioner's surgery or for training medicine students on orthopaedics.

Since the problem we are dealing with is highly complex and the dataset is of moderate size, we decided to apply a 47-cross validation protocol. The reason for this choice is that the number of subjects that suffer from knee OA is 47. This way, in each fold 46 subjects that have knee OA are used for training the knee OA multidimensional Gaussian model and the remaining one subject is used for testing. Accordingly, we maximise the number of subjects utilised for training. The accuracy achieved is 82.62±13.75% when utilising 36 PCs. The number of true positives across the trials equals 131, the number of false positives is 3, the number of false negatives is 35, and the number of true negatives equals 10. Thus specificity and sensitivity are 0.79 and 0.77 respectively; and precision is 0.97. The high number of false negatives proves that with this method we are able to recognise subjects that although they believe to be normal, in fact they exhibit motion patterns that are closer to those of those subjects that suffer from knee OA. It is reminded that we decided to use the aforementioned number of components since it explains about 99% of the variance.

## Discussion

### The complexity of human walking

In this paper we aim to investigate the fundamentals of human motion and how an understanding of this can be used to identify change in motion due to pathology, pain or other cause. Knee osteoarthritis is a condition we have applied to test the concept that complex data can be used to identify pathology or changes from normality, depicting the potential use of such analysis approach to assist in the diagnosis and management of complex clinical conditions. To achieve this goal, we applied machine learning. Machine learning concerns the construction of systems that can learn from data. It aims to reveal hidden patterns in the data and to build a system that performs well on unseen data instances.

We attempted to explore the complexity of human gait by projecting complex locomotion data to a low dimensional PC space. We take the view that the motor behaviour can be understood by identifying a simplicity, which may reflect upon the underlying mechanism [6], [7]. To extract the structure of gait cycle pattern variability we used PPCA which built generative models of walking and specifically, one model for normal walking and another one for that of subjects with knee OA. Importantly, the low-dimensional subspaces of just 36 dimensions appeared numerically distinct between subjects that suffer from knee OA and healthy ones. To quantify the complexity we proposed a novel complexity measure that has a tendency to be lower for knee OA subjects compared to normal ones for the first 7 PCs (except from the third PC where the complexity measure is almost equal for the two cases). This supports the view that the motor cortex organises behaviour in a low-dimensional manner to avoid the curse of dimensionality in terms of computational complexity. That is, it retains as much dimensions as needed for moving effectively, but not all the dimensions. All the dimensions include an enormous amount of information that would possibly impede movement. Additionally, it supports the hypothesis that specific walking patterns produce movement variability in characteristic sub-spaces. This is the reason that we are able to effectively predict the degree of knee OA by observing just a small amount of movement data (i.e. one to three steps).

Our remarks about the ability to model human motion in low PC spaces are in line with those presented in [33], where motion tracking is achieved by means of a Bayesian method, verifying the suitability of the Bayes theory to handle data that come from low-dimensional PC spaces. It is also interesting that the PC space has been proven suitable to capture different types of human activity by analysing data that come from hand movements [34], rather than from bipedal locomotion. Besides human motion, C. elegans motion is also effectively modelled by eigenanalysis that is related to PPCA [35].

### GRFs as predictors for knee OA

The structure of variability may contain valuable information about the way a task is performed [5] [36] and accordingly here the walking task may reveal osteoarthritic patterns. This is because it is expected that moving patterns are distorted in a systematic manner, rather than in a randon way, if a pathological factor is present. The latter is also verified from Figures 8a-8b, where the trajectories of the PCs for the normal and the pathological gait present differences that can be easily inspected visually. Even lower subspaces can effectively differentiate between normal and knee OA subjects. For example, if we confine ourselves to the 1-dimensional space the classification accuracy is 77.68±26.34%, for the same experimental protocol (i.e 47-folds cross-validated and subject-independent). This also suggests that GRFs are adequate predictors of knee OA. To support clinical decision making we propose a novel complexity measure that can be used as a possible indicator of knee OA. Another advantage of the proposed method is that it has a high number of false positives (also known as type I error), that is subjects that claimed not to have knee OA, but the proposed method estimates that their walking patterns are closer to osteoarthritic rather than the normal ones. An additional advantage of the proposed schema is that it can be easily transfered to other timeseries captured by alternative sensors during walking, such as accelerometers, gyroscopes, EMGs etc.

In fact, a range of techniques have been utilised in order to analyse data that come from normal and knee OA subjects, aiming to automatically differentiate between them. For example, in [37], wavelet analysis of GRFs has proved a reduction in peak anteriorposterior GRFs during the stance phase for knee OA subjects. Also, the vertical GRFs were lower in severe cases compared to the moderate cases. A total of 12 healthy and 24 knee OA subjects participated in the study. Wavelet transformation was also utilised by the authors of [38] to prove that the antero-posterior and medial-lateral force components in gait patterns carry the most discriminating power. The study included 16 healthy subjects and 26 subjects suffering a tibiofemoral knee OA. GRFs are also examined in [39], where subjects are asked to perform a sit-to-stand task. Twenty subjects with early medial knee OA and 20 control subjects participated in the study. It found that GRF integrals were significantly greater for knee OA sufferers. The advantage of our study is that it manges to visualise effectively the differences between the normal and the knee OA subjects. A very important aspect of this study is that, as it becomes obvious for Figures 8c-8d, it is the end of the stance phase that bears the most discriminating differences between the normal and the knee OA subjects. Specifically, in Figure 8c, it is easily seen that normal subjects have an extended stance phase, compared to knee OA subjects, since for the normal subjects PC values reach 0 after about 73% of the gait cycle, whereas for the knee OA subjects the latter value falls to about 71%. This is also verified from Figure 8d, where the most important PC differences between the two groups can be seen in the 45%–70% zone of the gait cycle. Accordingly, we propose that future studies may narrow down to this specific band of the gait cycle, rather than considering the whole gait cycle. The latter is expected to reduce the volume of the captured data, whereas at the same time reducing the non-informative parts of the gait cycle.

### Future work

Additional movements, namely stair ascent/descent, sitting and standing, and squat have been captured in our Lab and analysing those movements will enable us to discover which activities of daily life are mostly affected by knee OA. It will also help us verify that movements are organised in a low dimensional manner as well as rank them according to their complexity by specifying how many PCs are required by each type of movement in order to explain the movement's variability. The proposed framework for detecting pathology can be expanded to kinematic data. However, replicating the same protocol for another source of data, although expected to improve accuracy, falls out of the remit of this paper, since it would add unnecessary complexity. Since capturing GRFs requires a controlled environment, our next aim is the adaptation of our laboratory based computer tool to accommodate “GP based” measurements thereby enhancing clinical utility. For example, we could substitute the force plates with consumer balance boards such as Wii balance boards or with instrumented insoles. Further computational analysis will compensate for the errors introduced by those less accurate sensors. If the aforementioned system is portable, it could allow patients to self-manage, ensuring patient empowerment as well as enhancement of patient compliance with interventions. It could also be used as a novel diagnostic solution, that act prior to the patient feeling the need to go to see a clinician.

### Possible application to other areas

Other joints that suffer from OA, such as the hip, could benefit from the same type of analysis, since the underlying biomechanical mechanisms are the same. Facilitating diagnosis of alternative degenerative musculoskeletal conditions, such as carpal tunnel syndrome or back pain, could also benefit from the proposed approach. Additional problems where the neurological disorder affects the musculoskeletal system and causes impaired movement, such as Parkinson's disease or impaired locomotion due to strokes, could also benefit from the proposed framework.
